# Economic Evaluation of Dapagliflozin in the Treatment of Patients With Heart Failure: A Systematic Review

**DOI:** 10.3389/fphar.2022.860109

**Published:** 2022-04-14

**Authors:** Meiyu Wu, Shuxia Qin, Liting Wang, Chongqing Tan, Ye Peng, Xiaohui Zeng, Xia Luo, Lidan Yi, Xiaomin Wan

**Affiliations:** ^1^ Department of Pharmacy, The Second Xiangya Hospital, Central South University, Changsha, China; ^2^ Institute of Clinical Pharmacy, Central South University, Changsha, China; ^3^ PET Imaging Center, The Second Xiangya Hospital, Central South University, Changsha, China

**Keywords:** heart failure, dapagliflozin, cost-effectiveness analysis, systematic review, economic evaluation

## Abstract

**Objective:** The objective of this study is to systematically review the economic evaluations of dapagliflozin in the treatment of patients with heart failure (HF) and describe their general and methodological features.

**Methods:** This systematic review followed the PRISMA guidelines. MEDLINE/PubMed, Website Of Science, Embase, The Cochrane Library, ScienceDirect, CNKI, and Wanfang databases were searched to collect relevant studies, and the retrieval time ended on 31 October 2021. Articles on the economic evaluation of dapagliflozin in the treatment of heart failure were included. Secondary studies, incomplete economic indicators, and non-English-language and non-Chinese-language studies were excluded. Standard drug treatment was selected as the comparison. Basic characteristics, methods, and main results were extracted and analyzed systematically.

**Result:** A total of eight studies were identified, and the overall quality was accepted, which were performed in nine developed countries (Austria, United States, Korea, Japan, Singapore, Spanish, Germany, and United Kingdom) and three developing countries (the Philippines, Thailand, and China). With the exception of the Philippines, the remaining countries considered that dapagliflozin was cost effective. In the analyses of all included studies, the incremental cost-effectiveness ratios were most sensitive to the cost of dapagliflozin, cardiovascular mortality, the duration of dapagliflozin effectiveness, and the probability of HF hospitalization.

**Conclusion:** Dapagliflozin in the treatment of patients with heart failure with reduced ejection fraction was considered cost effective. Further studies are needed to evaluate the comprehensive value of dapagliflozin on HF.

## Introduction

Heart failure (HF) is one of the most common heart conditions and is associated with frequent hospitalization and high mortality (Roger et al., 2011; Gedela et al., 2015). China ranks first in the world in the number of HF patients (Bragazzi et al., 2021). Studies reported that approximately 12 million Chinese adults suffered from HF and estimated that the prevalence of HF would keep rising (Wang, 2017; Hao et al., 2019). In 2017, the estimated average annual hospitalization cost for HF was $4,406.8 per patient, and the cost of all drugs accounted for 30.6% of the total medical expenses (Wang et al., 2021). In recent years, several studies have proved the effectiveness of sodium-glucose cotransporter-2 (SGLT2) inhibitors in the treatment of patients with HF. In May 2020, dapagliflozin became the first SGLT2 approved by the United States Food and Drug Administration (FDA) for the treatment of HF patients with and without diabetes (Food and Drug Administ, 2020).

The clinical effects of the treatment of dapagliflozin for patients with type 2 diabetes mellitus (T2DM) are remarkable (Hwang et al., 2020; Tanaka et al., 2020). Studies reported that dapagliflozin treatment was cost effective for T2DM patients (Cai et al., 2019; Bennett et al., 2020). Currently, medical therapy for heart failure with reduced ejection fraction (HFrEF) has taken an important step (Kato et al., 2019; McMurray et al., 2019; Nassif et al., 2019; Petrie et al., 2020). The Dapagliflozin and Prevention of Adverse Outcomes in Heart Failure (DAPA-HF) trial has demonstrated the effects of dapagliflozin in patients with HFrEF by reducing HF hospitalization (HR 0.74, 95% CI 0.65–0.85) and cardiovascular (CV) mortality, including among patients without diabetes. Particularly, the results between patients with and without diabetes were similar in the DAPA-HF trial (McMurray et al., 2019; Petrie et al., 2020). In January 2021, dapagliflozin for HFrEF treatment in patients with and without diabetes was recommended by the American College of Cardiology Expert Consensus Decision Pathway for Optimization of HF Treatment (Maddox et al., 2021).

For the health care system to work better in the limitation of medical resources, the cost effectiveness of dapagliflozin in the treatment of patients with HF must be considered in clinical and policy decision-making. However, there is no systematic evaluation of the cost effectiveness of dapagliflozin in the treatment of HF. This study intends to provide a systematic review of the literature on pharmacoeconomics in the treatment of HF by dapagliflozin in order to provide support for clinical and policy decision-making in China.

## Materials and Methods

### Search Strategy

This systematic review followed The Preferred Reporting Systems for Systematic Reviews and Meta-Analysis (PRISMA). We used relevant keywords like “dapagliflozin,” “heart failure,” “economic,” “cost,” “cost-utility,” “cost-effectiveness,” and “cost-benefit” to search relevant studies in MEDLINE/PubMed, Website Of Science, Embase, The Cochrane Library, and ScienceDirect databases. The retrieval time ended on 31 October 2021. CNKI, Wanfang, and CBM databases were also used for searching the relevant literature published in Chinese. (Supplementary Table S1 provides the detailed search strategy used). We have not performed a review protocol nor registered the review prospectively.

### Inclusion and Exclusion Criteria

We have formulated the inclusion criteria according to the PICOs principle (Cumpston et al., 2019):

1. Population: patients with HF
2. Intervention: dapagliflozin
3. Comparison: standard drug treatment
4. Outcome: any outcomes of economic evaluation, such as life year saved (LYS), quality-adjusted life year (QALY), and incremental cost-effectiveness ratio (ICER)
5. Study design: basic pharmacoeconomic evaluation types, including cost-benefit analysis (CBA), cost-effectiveness analysis (CEA), and cost-utility analysis (CUA)

Studies meeting the following criteria were excluded:

1. Duplicated literature
2. No economic evaluation studies
3. Incomplete economic indicators, for example, researches that report only costs
4. The intervention was not dapagliflozin
5. Reviews, commentaries, letters, conference articles, and other secondary research
6. The language of the study was not English or Chinese
7. The full text of the article was not available

## Quality Evaluation

The quality of the included studies was accessed by two independent reviewers, using the checklist proposed by Drummond et al. (Drummond and Torrance, 2005). There are ten questions in the checklist, each with three possible responses (Yes, Unclear, and No), with 1 point for each ‘‘yes’’ response, and 0 point for each ‘‘No’’ response. Thus, the lowest and highest possible scores were 0 and 10 respectively. The quality of the study was classified as good if the score ≥7. Considering the risk of bias across studies, we checked for risk of bias across studies pertaining to clinical endpoints used. We have not assessed risk of bias of individual studies.

### Data Extraction and Analysis

We made tables to extract data from the literature for comprehensive analysis. Data extracted included basic information (i.e., name of the author, published year, country in which the study was performed, population, intervention, and comparison), perspective, time horizon, cost and effectiveness measures, types of economic model, discount rate, type of economic analysis, and the main results (e.g., total cost per patient, LYS, QALY, and ICER). The methods of uncertainty analysis were also recorded, generally including one-way sensitivity analyses and probabilistic sensitivity analyses (PSA). One-way sensitivity analysis estimates the impact of parameters by using the upper and lower limits of the parameter distributions to identify the most influential factors on ICER. We also performed a subgroup analysis based on the state of diabetes.

All reported ICERs in the included studies were transferred from the local currency in the year of the discount to the inflated values in local currency for the year 2020 (World Bank, 2021b). For better comparing the results of economic analysis between different currencies, we used the gross domestic product purchasing power parity (2020 PPP) to convert the ICERs data to United States dollars (USD) (World Bank, 2021a).

## Result

### Literature Search

**FIGURE 1:**
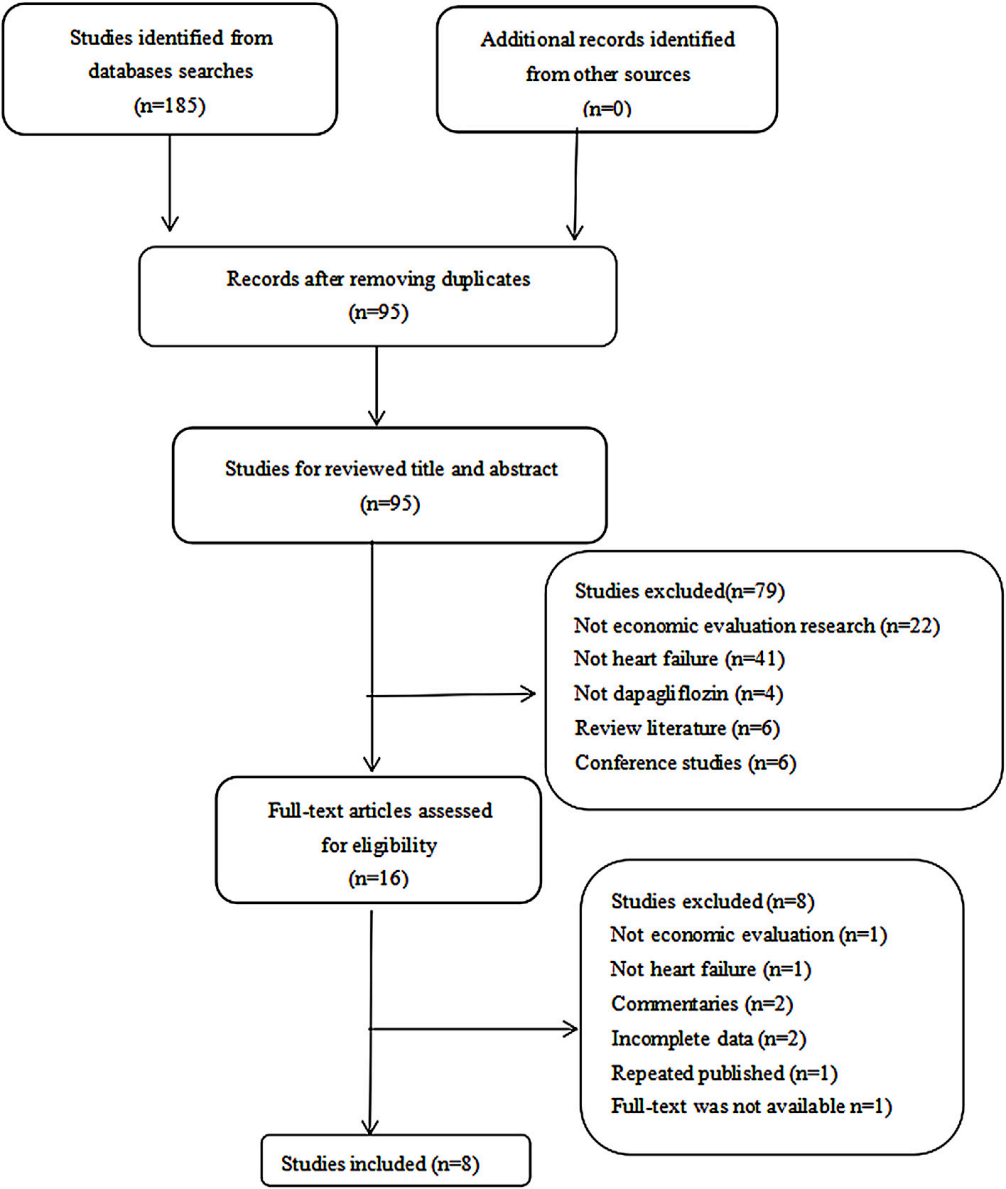
Diagram showing inclusion and exclusion of studies.

A total of 185 potentially relevant citations were retrieved on the initial search, and after moving 90 duplicates, the remaining 95 studies were screened by title and abstract. After excluding 79 articles as they did not meet the inclusion criteria, we selected 16 articles for a full-text screening. Eight articles were removed, primarily because they were commentaries (n = 2), no economic evaluation (n = 1), incomplete data (n = 2), not heart failure (n = 1), repeated publication (n = 1), and full text was not available (n = 1). Finally, eight articles were selected in this systematic review (McEwan et al., 2020; Yao et al., 2020; Isaza et al., 2021; Krittayaphong and Permsuwan, 2021; Liao et al., 2021; Mendoza et al., 2021; Parizo et al., 2021; Savira et al., 2021). More details of the retrieval process are shown in Figure 1.

### Quality Assessment of the Included Studies

**FIGURE 2:**
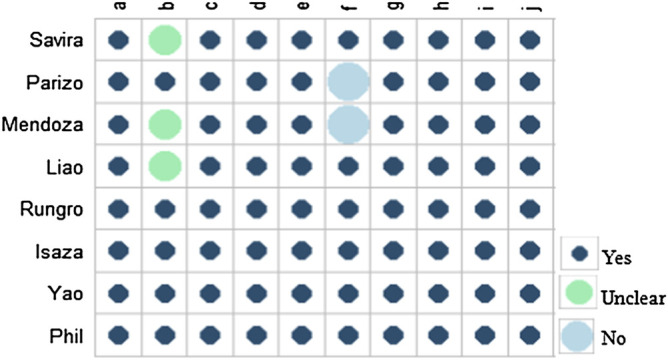
Quality assessment of the included studies. **(A)** Was a well-defined question posed in an answerable form? **(B)** Was a comprehensive description of the competing alternatives given? **(C)** Was the effectiveness of the programs or services established? **(D)** Were all the important and relevant costs and consequences for each alternative identified? **(E)** Were costs and consequences measured accurately in appropriate physical units? **(F)** Were costs and consequences valued credibly? **(G)** Were costs and consequences adjusted for differential timing? **(H)** Was an incremental analysis of costs and consequences of alternatives performed? **(I)** Was allowance made for uncertainty in the estimates of costs and consequences? **(J)** Did the presentation and discussion of study results include all issues of concern to the users?

Based on the Drummond checklist, all of the eight studies were identified as of good quality. The Drummond score of the included articles ranged from 8 to 10, with a median score of 9 (Drummond and Torrance, 2005). The results of the quality assessment are approved in Figure 2.

### Basic Characteristics

Eight articles selected in this review were published from 2020 to 2021, which were performed in nine developed countries (Austria, United States, Korea, Japan, Singapore, Spanish, Germany, and the United Kingdom) and three developing countries (Philippines, Thailand, and China). Markov models were used to evaluate the cost effectiveness in all included studies because they are especially suitable for modeling disease progression over time and can handle both the costs and effects at the same time (Briggs and Sculpher, 1998). The model simulated the population based on the characteristics of those in the DAPA-HF trial. In brief, the eligibility criteria of patients enrolled in the DAPA-HF trial included a left ventricular ejection fraction (LVEF) of 40% or less and the New York Heart Association (NYHA) class II, III, and IV symptoms (McMurray et al., 2019). Standard treatment was taken as the comparator, which was defined as the therapy for HFrEF based on the DAPA-HF trial. The standard treatment included an angiotensin-converting enzyme inhibitor (ACEI), an angiotensin receptor blocker (ARB) or sacubitril-valsartan plus a β-blocker. The intervention evaluated by the selected studies was dapagliflozin plus standard treatment. None of the included studies explicitly stated that economic evaluation report guidelines had been used.

The time horizon of most of the studies was a lifetime (n = 6) (McEwan et al., 2020; Isaza et al., 2021; Krittayaphong and Permsuwan, 2021; Mendoza et al., 2021; Parizo et al., 2021; Savira et al., 2021), and the remaining two studies were 15 years (Yao et al., 2020; Liao et al., 2021). Six studies used 1-year Markov cycles, and one study used 3-month Markov cycles, while one study however adopted 1-month Markov cycles. Half of the eight studies were performed using the perspective of health care (n = 4) (Isaza et al., 2021; Krittayaphong and Permsuwan, 2021; Liao et al., 2021; Savira et al., 2021), health care payer (n = 2) (Isaza et al., 2021; Parizo et al., 2021), health care provider (n = 1) (Parizo et al., 2021), or payers perspective (McEwan et al., 2020). None of the included studies was conducted from the social perspective.

### Cost and Health Outcomes

All eight studies evaluated the cost effectiveness in terms of direct costs in their researches, such as the cost of drug therapy, hospitalization, and death. Some articles also considered the cost of treating the adverse event, of which two studies comprised the costs of volume depletion and kidney injury (McEwan et al., 2020; Mendoza et al., 2021). We noticed that the annual costs of dapagliflozin varied in the selected articles (ranged from $257 to $5,683), partly because of the different countries in which the studies were set. The highest cost was from the United States ($4,192 per year), and the lowest one was from China ($257 per year) (Yao et al., 2020; Parizo et al., 2021). It is likely because Parizo et al. (Parizo et al., 2021) incorporated the total drug cost, including the dispensing fee, the drug plan payment, and the beneficiary co-payment. As a general rule, cost differences between studies may have an impact on the evaluated ICER.

QALYs were applied as a health outcome in all studies, which considered both the length and quality of life. Health utility values represented the quality of life for various health states, and the utility data of all studies were mainly from published primary and secondary researches of the DAPA-HF trial. The Kansas City Cardiomyopathy Questionnaire (KCCQ) was applied to assess the quality of life among DAPA-HF patients, which was thought to be suitable for the New York Heart Association (NYHA) Functional Class. LYS was also the main outcome measure.

In all selected studies, transition probabilities between different health states were based on the DAPA-HF trial, including HF hospitalization, CV mortality, non-cardiovascular (NCV) mortality, etc. However, it should be noted that clinical endpoints of included studies were all from one single trial, meaning that it may lead to the risk of lack of evidence in the literature due to publication bias. Furthermore, none of the included studies had considered additional country epidemiological data to adjust clinical endpoints derived from the trial. Six studies used a discount rate of 3% for costs and outcomes (McEwan et al., 2020; Isaza et al., 2021; Krittayaphong and Permsuwan, 2021; Liao et al., 2021; Mendoza et al., 2021; Parizo et al., 2021), whereas Savira et al. (Savira et al., 2021) and Yao et al. (Yao et al., 2020) used 5% and 4.2%, respectively. More details are shown in Table.1.

**TABLE 1:** Summary of included studies characteristics.

Author	Country	Disease	Intervention	Comparator	Age	Perspective	Model	Time	Length of cycle	Costs	Health outcomes	Sensitivity analysis
Savira et al., 2021 (Savira et al., 2021)	Australia	HFrEF	Dapa + sd	sd	66	Public healthcare system	Markov	Lifetime	1 year	1	LYS,QALY	1way,PSA
Parizo et al., 2021 (Parizo et al., 2021)	United States	HFrEF	Dapa + sd	sd	-	Healthcare payer	Markov	Lifetime	1 year	1	LYS,QALY	1way,2way,PSA
Mendoza et al., 2021 (Mendoza et al., 2021)	The Philippines	HFrEF	Dapa + sd	sd	66	Public healthcare provider	Markov	Lifetime	1 year	1	QALY	1way,PSA
Liao et al., 2021 (Liao et al., 2021)	Taiwan, China	HFrEF	Dapa + sd	sd	66	Healthcare system	Markov	15 years	1 month	1	QALY	1way,PSA
	Korea	HFrEF	Dapa + sd	sd	66	Healthcare system	Markov	15 years	1 month	1	QALY	1way,PSA
	Australia	HFrEF	Dapa + sd	sd	66	Healthcare system	Markov	15 years	1 month	1	QALY	1way,PSA
	Japan	HFrEF	Dapa + sd	sd	66	Healthcare system	Markov	15 years	1 month	1	QALY	1way,PSA
	Singapore	HFrEF	Dapa + sd	sd	66	Healthcare system	Markov	15 years	1 month	1	QALY	1way,PSA
Rungroj et al., 2021 (Krittayaphong and Permsuwan, 2021)	Thailand	HFrEF	Dapa + sd	sd	65	Healthcare system	Markov	Lifetime	1 year	1	LYS,QALY	1way,PSA

Author	Country	Disease	Intervention	Comparator	Age	Perspective	Model	Time	Length of cycle	Costs	Health outcomes	Sensitive analyze
Isaza et al., 2021 (Isaza et al., 2021)	United States	HFrEF	Dapa + sd	sd	66	Healthcare sector	Markov	Lifetime	1 year	1	LYS,QALY	1way,PSA
Yao et al., 2020 (Yao et al., 2020)	China	HFrEF	Dapa + sd	sd	65	Healthcare payer	Markov	15 years	3 months	1	LYS,QALY	1way,2way,PSA
Philet al. 2020 (McEwan et al., 2020)	Spain	HFrEF	Dapa + sd	sd	66	Payers	Markov	Lifetime	1 year	1	LYS,QALY	1way,PSA
	Germany	HFrEF	Dapa + sd	sd	66	Payers	Markov	Lifetime	1 year	1	LYS,QALY	1way,PSA
	United Kingdom	HFrEF	Dapa + sd	sd	66	Payers	Markov	Lifetime	1 year	1	LYS,QALY	1way,PSA

HFrEF, heart failure with reduced ejection fraction; DAPA, dapagliflozin; sd, standard treatment; LYS, life year saved; QALY, quality-adjusted life year; 1way, one-way sensitivity analyses; 2way, two-way sensitivity analyses; PSA, probabilistic sensitivity analyses; 1, direct cost.

### Economic Analysis

The majority of economic evaluations in this review both adopted CUA and CEA, while two studies only used CUA. All included articles have shown that the total cost per patient of dapagliflozin for HFrEF treatment was higher than the standard treatment, and the incremental cost ranged from $1,452 in China to $42,800 in the United States. However, the dapagliflozin made more benefit in terms of LYS and QALY, which result in that the incremental LYS ranged from 0.42 in Australia to 1.25 in Taiwan, China, and the incremental QALY ranged from 0.29 to 0.94. We extracted fourteen ICERs across eight studies, which pertained to twelve countries setting. To allow direct comparisons across countries, we converted all ICERs to 2,020 USD (based on the 2020 PPP), and the results ranged from $4,041 per QALY to $83,650 per QALY, the average value was $20,146 per QALY, and the median value was $13,738 per QALY. The lowest ICER was from China and the highest ICERs were from the United States (Yao et al., 2020; Parizo et al., 2021). We also found that the ICER from Thailand was followed by the lowest. But the ICER value of the Philippines was higher than some developed countries (Korea, Germany, and United Kingdom) and exceeded the Philippines’ willingness to pay (WTP) threshold (PHP180,500), which may due to the inadequacy of the distribution of medical services in the Philippines. More details are shown in Table.2.

**TABLE 2:** Methods and results of included studies.

Author	Country	Economic evaluation methods	Discount (cost | effect)	Total cost		LYS		QALY		△Cost	△LYS	△QALY
				A	B	A	B	A	B			
Savira et al., 2021 (Savira et al., 2021)	Australia	CEA,CUA	5% | 5%	$28,445,855	$24,753,415	4,047	3,631	2,789	2,502	$3,692,440	416	287
Parizo et al., 2021 (Parizo et al., 2021)	United States	CEA,CUA	3% | 3%	$183 583	$ 145,371	7.60	7.00	5.70	5.20	$38,212	0.60	0.50
Mendoza et al., 2021 (Mendoza et al., 2021)	The Philippines	CUA	3% | 3%	-	-	-	-	-	-	-	-	-
Liao et al., 2021 (Liao et al., 2021)	Taiwan, China	CEA,CUA	3% | 3%	$87,805	$76,501	14.71	13.46	11.03	10.09	$11,304	1.25	0.94
	Korea	CUA	3% | 3%	$17,577	$13,277	-	-	9.56	8.74	$4,300	-	0.82
	Australia	CUA	3% | 3%	$59,126	$50,745	-	-	9.85	9.01	$8,381	-	0.84
	Japan	CUA	3% | 3%	$49,064	$35,453	-	-	9.56	8.74	$13,611	-	0.82
	Singapore	CUA	3% | 3%	$160,525	$140,153	-	-	10.29	9.42	$20,372	-	0.87
Rungroj et al., 2021 (Krittayaphong and Permsuwan, 2021)	Thailand	CUA	3% | 3%	THB54,405	THB17,442	10.23	9.35	6.92	6.33	THB36,963	0.88	0.59
Isaza et al., 2021 (Isaza et al., 2021)	United States	CEA,CUA	3% | 3%	$193,400	$150,600	6.60	5.91	5.36	4.73	$42,800	0.69	0.63
Yao et al., 2020 (Yao et al., 2020)	China	CEA,CUA	4.2% | 4.2%	$5,829	$4,377	7.11	6.60	4.82	4.44	$1,452	0.51	0.38
Phil et al., 2020 (McEwan et al., 2020)	Spain	CEA,CUA	3% | 3%	€24,330	€19,642	6.35	5.74	4.70	4.22	€4,688	0.61	0.48
	Germany	CEA,CUA	3% | 3%	€25,328	€22,647	6.35	5.74	4.72	4.22	€2,681	0.61	0.50
	United Kingdom	CEA,CUA	3% | 3%	£16,408	£13,628	6.20	5.62	4.61	4.13	£2,780	0.58	0.48

Author	Country	Discount year	ICER		Local currency in 2020	ICER, USD (PPP 2021)	WTP	Funding source
			/LYS	/QALY	/QALY	/QALY		
Savira et al., 2021 (Savira et al., 2021)	Australia	2020	$8,875	$12,842	$12,842	$12,842	$50,000	Monash International Postgraduate Research Scholarship; Monash Graduate Scholarship
Parizo et al., 2021 (Parizo et al., 2021)	United States	2020	$68,819	$83,650	$83,650	$83,650	$50,000∼$150,000	National Heart, Lung, and Blood Institute of the National Institutes of Health
Mendoza et al., 2021 (Mendoza et al., 2021)	The Philippines	2019	-	PHP188,450	PHP193,406	$9,913	PHP180,500	AstraZeneca Pharmaceuticals (Phils)
Liao et al., 2021 (Liao et al., 2021)	Taiwan, China	2020	$9,080	$12,305	$12,305	$12,305	$25,000∼$75,000	Ministry of Science and Technology in Taiwan
	Korea	2020	-	$5,277	$5,277	$5,277	$30,000	
	Australia	2020	-	$9,980	$9,980	$9,980	$52,000	
	Japan	2020	-	$16,705	$16,705	$16,705	$39,000	
	Singapore	2020	-	$23,227	$23,227	$23,227	$58,000	
Rungroj et al., 2021 (Krittayaphong and Permsuwan, 2021)	Thailand	2019	THB42,173	THB62,090	THB61,565	$4,990	THB160,000	-
Isaza et al., 2021 (Isaza et al., 2021)	United States	2020	$61,800	$68,300	$68,300	$68,300	$50,000∼$150,000	Richard A. and Susan F. Smith Center for Outcomes Research in Cardiology
Yao et al., 2020 (Yao et al., 2020)	China	2017	$2,809	$3,827	$4,041	$4,041	$8,773	Key Projects in the National Science and Technology Pillar Program of the 13th Five‐Year Plan Period
Phil et al., 2020 (McEwan et al., 2020)	Spain	2019	-	€ 9,406	€ 9,375	$15,172	€20,000	AstraZeneca Pharmaceuticals (Phils)
	Germany	2019	-	€ 5,379	€ 5,406	$7,252	€20,000	
	United Kingdom	2019	-	£5,822	£5,879	$8,399	£20,000	

CEA, cost-effectiveness analysis; CUA, cost-utility analysis; LYS, life year saved; QALY, quality-adjusted life year; △Cost, increment cost; △LYS, increment life year saved; △QALY, increment quality-adjusted life year; A, intervention; B, comparator, ICER, increment cost-effectiveness ration; PPP, purchasing power parity; WTP, willingness to pay.

Of the treatment regimens reviewed, treating HF patients with dapagliflozin was identified to be cost effective except by Mendoza et al. (Mendoza et al., 2021). In the Philippines, add-on dapagliflozin in HFrEF patients was not considered to be cost effective (with the ICER of PHP188450 per QALY) when the unit cost of a dapagliflozin 10 mg tablet was PHP46.50. However, the dapagliflozin would be cost effective with the ICER of PHP177868 per QALY gained if the unit cost was PHP44.00, and it would be more cost effective for HFrEF patients with diabetes if the unit cost was PHP40.00, resulting in the ICER of PHP120249 per QALY.

### Uncertainty Analysis

One-way sensitivity analyses and PSA were applied in all included studies. Two studies applied two-way sensitivity analyses on the basis of the preceding two sensitivity analyses (Yao et al., 2020; Parizo et al., 2021). Six studies reported that the ICERs were mainly influenced by the cost of dapagliflozin (Yao et al., 2020; Isaza et al., 2021; Krittayaphong and Permsuwan, 2021; Liao et al., 2021; Mendoza et al., 2021; Parizo et al., 2021), Yao et al. (2020) found that the ICER ($12,696 per QALY gained) was higher than the per capita Gross Domestic Product for China in 2017 ($8,573) when the cost of dapagliflozin was at its upper limit, which made the cost of dapagliflozin become the most influential factor on the ICER. Mendoza et al. (2021) showed that the dapagliflozin in treatment for patients was not cost effective when the unit cost of dapagliflozin was PHP46.50, but it would be cost effective when the cost was PHP44.00.

Five studies displayed that the CV mortality also had a great impact on ICERs (Yao et al., 2020; Krittayaphong and Permsuwan, 2021; Liao et al., 2021; Mendoza et al., 2021; Parizo et al., 2021). With the decrease of CV mortality of standard treatment or the increase of CV mortality of dapagliflozin, the ICER gets higher. Parizo et al. (2021) showed that across the 95% CI (0.69–0.98) for HR of CV mortality, the ICER ranged from $58,747 to $361,739. To meet the WTP threshold of $50,000 per QALY and $150,000 per QALY, the HR for CV mortality with dapagliflozin would need to be less than 0.59 and 0.92, respectively. Liao et al. (2021) displayed that with the CV mortality of dapagliflozin increased from 0.496 to 0.607%, and the ICER increased from $10,247 per QALY gained to $15,297 per QALY gained. However, when the CV mortality of dapagliflozin increased from 0.603 to 0.737%, the ICER decreased from $16,442 to $10,534.

Three studies also displayed the ICERs were sensitive to the duration of dapagliflozin effectiveness (Isaza et al., 2021; Liao et al., 2021; Parizo et al., 2021). One study revealed that the key driver of cost effectiveness was the probability of HF hospitalization (Savira et al., 2021). However, in the sensitivity analysis of all included articles, varying parameters were based on credible intervals reported in the trial or a specific range. For example, in the work of Rungroj et al., all probabilities were varied within the range of ±10% when performing an univariate sensitivity analysis.

### Subgroup Analysis

Subgroup analysis was performed in five studies (Isaza et al., 2021; Krittayaphong and Permsuwan, 2021; Mendoza et al., 2021; Parizo et al., 2021; Savira et al., 2021), revealing that the ICERs in diabetes was similar to without diabetes, and dapagliflozin was more cost effective in HErEF patients with diabetes. However, Savira et al. (2021) demonstrated that dapagliflozin was more cost effective in subjects with diabetes only when the time horizon was limited to 2 years, with the ICER of $32,098 per QALY (with diabetes) compared with $42,178 (without diabetes). More details are shown in Table.3.

**TABLE 3:** Subgroup analyses of diabetes status.

Authors	Country	/LYS		/QALY	
		With diabetes	Without diabetes	With diabetes	Without diabetes
Savira et al., 2021 (Savira et al., 2021)	Australia	$9,148	$8,847	$12,605	$12,386
Parizo et al., 2021 (Parizo et al., 2021)	United States	$63,844	$71,456	$79,726	$85,420
Mendoza et al.202 (Mendoza et al., 2021)	The Philippines	-	-	PHP140,290	PHP295,131
Rungroj et al., 2021 (Krittayaphong and Permsuwan, 2021)	Thailand	THB32,302	THB46,420	THB47,613	THB68,304
Isaza et al., 2021 (Yao et al., 2020)	United States	$57,300	$66,200	$66,800	$69,600

## Discussion

This study systematically reviewed the pharmacoeconomics evaluations of dapagliflozin in the treatment of patients with HF worldwide, where it turns out that dapagliflozin is cost effective in most countries. All reported ICERs of the included studies were converted to USD (based on the PPP, 2020), which was shown that ICERs vary greatly from study to study. It is mainly because of the difference in medical and economic levels in different countries. With the exception of the Philippines, the remaining countries considered that dapagliflozin in the treatment of patients with HFrEF was cost effective. It means that conclusions on the cost effectiveness of drugs in one country cannot be applied to another. Barbieri et al. (2005) already demonstrated the variability of cost-effectiveness estimates for pharmaceuticals in Western Europe. Liu et al. (2021) identified that sacubitril-valsartan for heart failure was supposed to be cost effective in the United States and other developed countries, but not in Thailand.

It is worth noting that although the studies came from the same country, the results were still different. The ICERs of two Chinese studies varied greatly. One came from Taiwan, China, with the ICER of $12,305 per QALY (Liao et al., 2021), and the ICER of another was $4,041 per QALY (Yao et al., 2020). Due to the differences between the two regions in terms of economic level and public health policies, we should consider the heterogeneity in different regions of China when we evaluate the cost effectiveness of dapagliflozin in treatment for HF patients.

The reported ICERs from two United States-based studies varied ($83,650 per QALY vs. $68,300 per QALY) (McEwan et al., 2020; Parizo et al., 2021). It is likely because the former applied a higher cost of dapagliflozin ($5,683 per year), but the latter used a lower cost of dapagliflozin ($4,192 per year). The cost of dapagliflozin was the dominant parameter affecting ICERs. Isaza et al. (2021) demonstrated that the ICER for dapagliflozin compared with the standard treatment alone would decline to $29,400 per QALY if the annual cost of dapagliflozin could be reduced to $500. Four studies (Yao et al., 2020; Krittayaphong and Permsuwan, 2021; Liao et al., 2021; Mendoza et al., 2021) also confirmed that the cost of dapagliflozin played an important role in impacting ICERs. Dapagliflozin in treatment for HErEF patients will be more cost effective if a lower price of dapagliflozin is negotiable.

Among ICERs of the countries involved in the studies, the lowest ICER came from China, but it did not imply that the use of dapagliflozin in China was the most cost-effective. The cost is the main driver when we evaluate whether a treatment is cost effective or not. Costs of treating HF patients with dapagliflozin between China and developed countries involved in the studies were different, which is mainly due to the difference between China and developed countries in terms of the economic level and medical level. With the development of China’s economy, Chinese citizens have higher requirements for quality of life. At present, the evidence of the cost effectiveness of dapagliflozin in the treatment of patients with HF in China is limited. Considering the diversity and specificity of the population of China, further evaluation of the cost effectiveness of dapagliflozin in China is necessary.

Furthermore, we need to pay special attention that the models of all included studies were based on the same trial, which means that the results may be affected by publication bias, that is, most articles tend to publish positive studies. This may have a significant impact on the reliability of the research outcomes. Although all the included studies had performed sensitivity analyses, the variation range of some parameters was still empirical. Only relying on experience for sensitivity analysis is likely to have a critical impact on the reliability and authenticity of the results. None of the eight included studies applied CBA, and the absence of CBA studies which does not provide evidence on other non-health parameters possibly valued by patients.

### Limitation

Several limitations in this review must be acknowledged. First, we did not search for other electronic sources and unpublished studies. Second, the language was limited to English or Chinese, which may result in information bias and missing some relevant studies. But we used several online databases that may minimize the impact. Third, data concerning the cost effectiveness of dapagliflozin in treatment for HFrEF patients in China remain insufficient and the studies included in this review involved twelve countries. Because outcomes are different worldwide, extrapolating the data reviewed in China may have some limitations. Fourth, all economic studies included in this review used clinical endpoints derived from the same trial, which may affect the reliability of the results because of publication bias. Fifth, we acknowledge that we used an older instrument for critical appraisal of economic studies, instead of the Consolidated Health Economic Evaluation Reporting Standards (CHEERS) scale.

## Conclusion

In summary, add-on dapagliflozin for patients with HFrEF not only increased the total cost but also prolonged the lifetime of patients and improved the quality of life. Based on the included studies in this review, dapagliflozin in the treatment of patients with HFrEF was considered cost effective. The ICERs were most sensitive to the cost of dapagliflozin, CV mortality, the duration of dapagliflozin effectiveness, and the probability of HF hospitalization. Further economic evaluations of dapagliflozin on heart failure need to take into account the country epidemiological real-world data in relevant input parameters in the sensitivity analysis. In order to understand the preferences for other non-health patients, exploring the cost-benefit studies on this technology and population in future is necessary.

## Data Availability

The original contributions presented in the study are included in the article/Supplementary Material, further inquiries can be directed to the corresponding author.
