# Supplementary material for: Economic Evaluation of Dapagliflozin in the Treatment of Patients With Heart Failure: A Systematic Review
Source: Front Pharmacol. 2022 Apr 14;13:860109. doi: 10.3389/fphar.2022.860109 (PMC9046576; doi:10.3389/fphar.2022.860109)
Supplement: Supplementary file 1 [file Table1.DOCX]

**Table S1 Search strategy used**

| **Database** | **search strategy** |
| --- | --- |
| MEDLINE/PubMed | (((((economic) OR (cost_effectiveness)) OR (cost_benefit)) OR (cost_utility)) AND (heart failure)) AND (dapagliflozin) |
| Embase | 'heart failure' AND 'dapagliflozin' AND ('economic' OR 'cost-effectiveness' OR 'cost-benefit' OR 'cost-utility') |
| The Cochrane Library | heart failure in Title Abstract Keyword AND economic OR cost_effectiveness OR cost analysis OR cost_benefit in Title Abstract Keyword AND dapagliflozin in Title Abstract Keyword - (Word variations have been searched) |
| Website Of Science | ((ALL=(dapagliflozin)) AND ALL=(heart failure)) AND ALL=(economic OR cost_effectiveness OR cost_utility OR coat_benefit OR cost) |
